# Acute Cardiorespiratory and Metabolic Responses to Incremental Cycling Exercise in Endurance- and Strength-Trained Athletes

**DOI:** 10.3390/biology11050643

**Published:** 2022-04-22

**Authors:** Maciej Jurasz, Michał Boraczyński, James J. Laskin, Anna M. Kamelska-Sadowska, Robert Podstawski, Jarosław Jaszczur-Nowicki, Jacek J. Nowakowski, Piotr Gronek

**Affiliations:** 1 Department of Sport Medicine and Traumatology, Poznań University of Physical Education, 61-871 Poznań, Poland; maciej@archeus.pl; 2 Faculty of Health Sciences, Collegium Medicum, University of Warmia and Mazury in Olsztyn, 10-719 Olsztyn, Poland; 3 School of Physical Therapy and Rehabilitation Sciences, University of Montana, Missoula, MT 59812, USA; james.laskin@mso.umt.edu; 4 Department of Rehabilitation and Orthopedics, School of Medicine, Collegium Medicum, University of Warmia and Mazury in Olsztyn, 11-082 Olsztyn, Poland; kamelskamedical@gmail.com; 5 Clinic of Rehabilitation, Provincial Specialist Children’s Hospital in Olsztyn, 10-561 Olsztyn, Poland; 6 Department of Tourism, Recreation and Ecology, University of Warmia and Mazury in Olsztyn, 10-957 Olsztyn, Poland; podstawskirobert@gmail.com (R.P.); j.jaszczur-nowicki@uwm.edu.pl (J.J.-N.); 7 Department of Ecology and Environmental Protection, University of Warmia and Mazury in Olsztyn, 10-727 Olsztyn, Poland; jacek.nowakowski@uwm.edu.pl; 8 Laboratory of Healthy Aging, Department of Dance, Poznań University of Physical Education, 61-871 Poznań, Poland; gronek@awf.poznan.pl

**Keywords:** cardiometabolic parameters, progressive cycle performance, triathlon athletes, resistance training, bodybuilders

## Abstract

**Simple Summary:**

The multiple structural and functional adaptive changes vary depending on the mode of training. The purpose of this study was to examine the acute effects of progressive submaximal cycling on selected cardiorespiratory and metabolic variables in endurance- and strength-trained athletes. The sample comprised participants with different training background: endurance trained group (triathletes), strength trained group (bodybuilders), and a control group (recreationally active students). The research problem was based on the verification of the minor non-specific cardiometabolic responses in strength and endurance trained athletes during a progressive submaximal cycling exercise test. The substantive finding of this study was that endurance- and strengthtrained athletes differed only in the metabolic responses of respiratory exchange ratio and blood lactate concentration, whereas the acute cardiorespiratory variables did not demonstrate any statistically relevant differences. Based on our findings we recommend that endurance-trained athletes follow a concurrent training program, combined strength and endurance training, to improve neuromuscular parameters and thus optimize their economy of movement and endurance-specific muscle power capacity.

**Abstract:**

The purpose of this study was to examine the acute effects of a progressive submaximal cycling exercise on selected cardiorespiratory and metabolic variables in endurance and strength trained athletes. The sample comprised 32 participants aged 22.0 ± 0.54 years who were assigned into three groups: an endurance trained group (END, triathletes, *n* = 10), a strength trained group (STR, bodybuilders, *n* = 10), and a control group (CON, recreationally active students, *n* = 12). The incremental cycling exercise was performed using a progressive protocol starting with a 3 min resting measurement and then a 50 W workload with subsequent constant increments of 50 W every 3 min until 200 W. The following cardiometabolic variables were evaluated: heart rate (HR), oxygen uptake (VO_2_), carbon dioxide production (VCO_2_), respiratory exchange ratio (RER), systolic and diastolic blood pressure (SBP and DBP), and blood lactate (BLa^−^). We found the between-group differences in metabolic variables (the average RER and BLa^−^) were statistically significant (Tukey’s HSD test: CON vs. STR, *p* < 0.01 and *p* < 0.05, respectively; CON vs. END, *p* < 0.001; END vs. STR, *p* < 0.001). RER and BLa^–^ differences in all groups depended on the workload level (G-G-epsilon = 0.438; *p* < 0.004 and G-G-epsilon = 0.400; *p* < 0.001, respectively). There were no significant differences in cardiorespiratory variables between endurance- and strength-trained groups. In conclusion, this study demonstrated that acute cardiorespiratory responses at each of the four submaximal workloads were comparable in endurance- compared to strength-trained athletes, but significantly different compared to recreationally active men. However, there were significant differences in the metabolic responses of RER and BLa^−^. Based on our findings we recommend that endurance-trained athletes follow a concurrent training program, combined strength and endurance training, to improve neuromuscular parameters and thus optimize their economy of movement and endurance-specific muscle power capacity.

## 1. Introduction

Multiple structural and functional adaptive changes vary depending on the mode of training—cardiovascular endurance versus muscular strength. Endurance training maximizes the athletic performance in prolonged endurance efforts (>30 min) that involve large muscle masses (low-resistance high-repetition exercises) and is performed at submaximal power outputs or intensities such as a percentage of maximal oxygen consumption (%VO_2max_). The specific physiological adaptations of endurance training in the cardiorespiratory system include: increased aerobic capacity, a decrease in heart rate (HR) at rest and, during any given submaximal effort, a decrease or stabilization of blood pressure (BP), an increase in stroke volume (SV) at rest and during submaximal exercise, an increase in the maximal cardiac output (CO) and ejection fraction (EF), as well as a decrease in overall systemic vascular resistance (SVR_peak_) [1,2]. For the endurance-trained athletes, (ETA) who undertake activities such as long-distance running, road cycling, cross-country skiing, and rowing, the volumetric changes in the circulatory system are characterized by the increased left ventricular end-diastolic volume, muscle mass, and cavity dimensions [3]. Moreover, specific methods of overload endurance training are associated with shorter post-exercise heart rate recovery (HRR) and a faster return of physiological variables to the pre-exercise (baseline) levels in the well-trained triathlete [4]. A recent study indicated that during rowing (at a power-to-weight ratio of 2 W∙kg^–1^ for 2 min), the increase in SV and CO was greater than in protocol-matched cycling exercise, whereas HR and BP were not influenced by the type of exercise [5]. 

In contrast, resistance training (high-resistance, low-repetition exercises) results in the skeletal muscle adaptations such as increased force output [6], muscle hypertrophy [7] and, possibly, hyperplasia [8]. Short- and long-term studies of highly strength-trained athletes (STA) indicate that resistance training, specifically continuous and prolonged circuit resistance training, enhances the toleration of physiological environments where high cardiovascular demands and higher lactate concentrations are present [9]. While cardiovascular variables, such as systolic BP (SBP), diastolic BP (DBP), and HR, are significantly lower in ETA compared to STA and sedentary healthy individuals [10], it has been reported that strength training causes a lower rise in CO, a marked increase in BP, and concentric remodeling of the heart with HR within the normal range [10,11]. However, the strength training mode is crucial to the morphological and functional adaptations because bodybuilders, but not weightlifters, have greater diastolic and systolic right ventricular internal dimensions at rest in absolute and relative to body surface area and lean body mass terms [12]. Moreover, the post-exercise HRR accelerates faster in STA compared to ETA [12]. From a metabolic perspective, resistance training should stimulate the aerobic system; however, many researchers conclude that this training modality is not the appropriate stimulus to significantly improve aerobic metabolism [13,14]. In addition, even when an increase in VO_2peak_ is elicited by strength training, the increase demonstrated is substantially lower than the 15–25% increases associated with traditional cycling, running, and swimming endurance training programs [15,16]. Most importantly, the concurrent training effect or interference effect as a combination of the latter mentioned two types of exercise (endurance and resistance training) may provide more comprehensive cardiovascular disease benefits compared to time-matched aerobic or resistance training alone [17].

It is generally accepted by coaches and sports scientists that endurance and strength training modes of exercise, when repeated over time, elicit distinct and competing adaptive mechanisms, including the genetic and molecular mechanisms of adaptation, that generate the specific exercise induced phenotype associated with long-term training. However, many factors, such as the volume and specifically the intensity of the training program, may influence to what extent any adaptation occurs, aerobic or otherwise [18]. Indeed, similar adaptations to high-intensity interval training are likely to occur via resistance/strength training to volitional failure. These observations suggest that stress to the cardiovascular system is crucial, and the modality of exercise may be irrelevant to the stimulation of aerobic metabolic pathways [19]. Due to inconsistent data regarding the exercise-induced specific adaptation responses in resistance-only trained and endurance-only trained athletes, the purpose of this study was to examine the acute effects of progressive submaximal exercise on selected cardiorespiratory and metabolic variables in endurance and strength trained athletes. In other words, we evaluated the integrated cardiorespiratory and metabolic responses in asymptomatic athletes with different training backgrounds to confirm and potentially supplement previously reported specific exercise-induced adaptations at submaximal efforts. The research problem was designed to verify previously reported minor non-specific cardiometabolic symptoms in strength- and endurance-trained athletes during a progressive cycling exercise test. It was hypothesized that the training-dependent adaptations would provoke significantly different cardiometabolic responses. Specifically, it was assumed that individuals who were endurance-trained would perform a progressive submaximal cycling test with lower acute cardiorespiratory and metabolic responses despite a relatively high peak workload intensity as compared to those who are recreationally active or strength-trained.

## 2. Materials and Methods

### 2.1. Participants and Eligibility Criteria

A total of 47 male participants were recruited from various gyms and sports organizations across the western region of Poland via email and recruitment flyers. Due to COVID-19 restrictions, a direct recruitment process (face to face) was not possible. Thirty-two participants (mean ± SD: age 22.0 ± 0.54 years, age range 19–25) met the three specific group criteria for this cross-sectional observational study. The three groups were: (1) an endurance-trained group (END, *n* = 10), consisting of regionally and nationally ranked triathletes with 4.6 ± 0.72 years competitive experience at the short Olympic distance triathlon performance (i.e., 1.5 km swimming; 40 km cycling; 10 km running); (2) a strength-trained group (STR, *n* = 10), consisting of natural (drug-free) bodybuilders with 4.2 ± 0.52 years competitive experience in bodybuilding performance; and (3) a control group (CON, *n* = 12), consisting of recreationally-active non-athletes who performed regular moderate-to-vigorous exercise 2–3 times per week, but did not train or compete within a given sport at a competitive level. The characteristics of all participants are presented in Table 1.

Inclusion criteria for END and STR groups included: male, aged 18–30 years, self-reported regular engagement in endurance or strength training, at least 3 years of competitive experience, no current sustained experience in the other modes of training, non-smokers, and no reported medical contraindications to participate in exercise testing. For the CON group, the inclusion criteria included: male, aged 18–30 years, non-smokers, no reported medical contraindications to participate in exercise testing, a BMI < 25 kg/m^2^ and active lifestyle (>600 MET) confirmed by an adapted version of short-form of International Physical Activity Questionnaire (IPAQ-SF) (http://www.ipaq.ki.se/, accessed on 7 October 2020).What should be indicated here is that the recruitment of recreationally active participants (>600 MET/week) was made on purpose to confirm that athletes from END and STR groups had specific exercise-induced adaptations.

The participants in the END group reported that during the past 12 months their weekly training volume averaged 14.5 ± 1.3 h, with 1–2 daily exercise sessions in each of the threetriathlondisciplines (4.9 ± 0.3 h for swimming, 5.7 ± 0.6 h for cycling, 3.1 ± 0.3 h for running, and 0.8 ± 0.1 h for specific preparation). The participants reported an intensity during any given training session of 15–17 on the Borg Rating of Perceived Exertion scale (RPE). 

Those in the STR group averaged 11.9 ± 0.6 h of training per week with 5.1 ± 1.3 sessions per week (2.4 ± 0.3 h/session) and reported an average training intensity of 15–16 RPE. The strength training programs consisted of multi-joint exercises that promoted sufficient stimuli for gaining muscle mass and strength in all muscles involved in the exercise. They typically performed 2–3 sets of 10–12 exercises with each set consisting of 6–12 repetitions.

Participants in the CON group reported engaging in a variety of types of activities including vigorous (e.g., heavy lifting, treadmill running); moderate (e.g., carrying light loads, cycling); and walking activities. Based on their IPAQ-SF scores they were classified as moderately active (≥600 MET–minutes/week). The CON group’s mean total MET score was 954 ± 287.3 MET–minutes/week.

### 2.2. Ethical Approval

All participants provided their written informed consent and verbal assent regardingtheir participationin the study. The research and protocol were approved by the Ethics Committee of the Karol Marcinkowski University of Medical Sciences in Poznań, Poland. All the procedures performed in this study conformed to the ethical guidelines of the 2013 World Medical Association Declaration of Helsinki (Fortaleza, Brazil).

### 2.3. Design and Procedures

Data collection was performed over a 2-week period, in which participants took part in laboratory tests on two separate occasions with a minimum of 48 h between sessions. During the first visit to the laboratory, anthropometric data were collected, and all participants underwent a non-invasive pre-participation cardiovascular screening (based on 12-lead ECG). During the second visit, participants had a familiarization session (cycle ergometer, gas analyzer and the testing procedure) and performed the incremental cycling test. The incremental cycling test was conducted in the morning hours because of the daily variability in variables measured in this study. Participants performed tests in a condition-controlled laboratory room with a fan (average temperature: 22.5 ± 0.5 °C, relative humidity: 35.0 ± 5.0%, barometric pressure: 760–770 mmHg).

### 2.4. Pre-Test Preparation

Participants were required to refrain from strenuous exercise during the 48 h prior to the incremental cycling test session. No alcohol, caffeine ortobacco were allowed for 24 h prior to testing. In addition, participants were asked to avoid using any ergogenic aids for at least 48 h before the testing session. They were also instructed to refrain from eating for 2 h prior to testing and to only drink water. Participants were asked to visit the laboratory at a similar (±1 h) time of a day to minimize any diurnal effects and circadian rhythms.

### 2.5. Anthropometry

Body height (BH) was measured to the nearest 0.1 cm using a patient weighing scale with aheight rod (Seca 217, Hamburg, Germany). Body mass (BM, after removal of shoes and heavy clothing) was measured to the nearest 0.1 kg.

### 2.6. Incremental Cycling Test

The exercise test was performed using the electromagnetically braked cycle ergometer (Ergometrics 900 S, Ergo-line GmbH, Bitz, Germany). Before the study, the cycle ergometer was calibrated for power outputs of 25–1000 W at different cadences and was found to be within 1% of a true value. The cycle ergometer was set according to each participant’s anatomy;the participant adjusted saddle height and the handlebars to his own cycling posture. However, the trunk forward inclination angle did not exceed 20°, and the knee flexion angle was 5° with the extended lower limb. A three-minute standardized warm-up protocol was followed prior to testing. The latter was performed at a constant workload (1.5–2.0 W∙kg^–^^1^ of BM) and pedaling cadence (70 rotations∙min^−1^) interspersed with 2 all-out sprints of 2–3 s (~90 rotations∙min^−1^) to elicit an HR between 150 and 160 beats∙min^−1^. Participants then rested for 15 min prior to the incremental cycling test. This graded test was performed using a progressive protocol according to Dufour et al. [20]. The test started with a 3min resting measurement in the sitting position and then a 50 W workload with 3 subsequent increments of 50 W every 3 min (100, 150 and 200 W). The cadence was set at 70 rotations∙min^−1^ and was maintained via the use of a metronome.

### 2.7. Cardiorespiratory Measurements

Respiratory gas data werecollected continuously by use of an automated breath-by-breath computer-based metabolic system (CPX/D, MedicalGraphicsCorp, St. Paul, MN, USA). The validation of the CPX/D under both normoxic and moderate hyperoxic conditions was shown previously [21]. The measuring device was calibrated before the exercise test according to the manufacturer’s instructions using reference and calibration gases, and the necessary environmental adjustments were made. During the incremental cycling test, the following cardiorespiratory variables were obtained: heart rate (HR, beats∙min^–^^1^), oxygen uptake (absolute VO_2_, L∙min^–^^1^ and relative VO_2_, mL∙min^–^^1^∙kg^–^^1^), carbon dioxide production (VCO_2_, L∙min^–^^1^), and respiratory exchange ratio (RER). Peak VO_2_, VCO_2_ and RER were expressed as the highest averaged samples, mean from the last 30 s, obtained during each of the four 3-min workloads. Breath-by-breath VO_2_ data from bouts of exercise were initially examined to exclude errant breaths caused by coughing, swallowing, sighing, etc. Any values during the 30 s data collection lying from more than four SDs from the mean were removed. Heart rate was continuously recorded (at 5-s intervals) throughout the whole exercise test using the portable heart rate monitor (Sport Tester PE 3000, Polar Electro Oy, Kempele, Finland). The SBP and DBP were automatically and non-invasively measured during every stage of the exercise test with a cuff-wrist sphygmomanometer HEM-6000 (OMRON, Osaka, Japan). The cuff was applied to the right wrist and aligned with the same level of heart during BP measurement and recoded every minute.

### 2.8. Biochemical Analysis

Blood lactate concentration (BLa^–^, mmol∙L^–1^) was assessed using the enzymatic method according to Gutmann and Wahlefeld [22] with the use of Boehringer reagents (Mannheim, Germany). Blood samples (4 mL) were taken from the basilic vein via a catheter inserted half an hour before the start of the exercise test, pre-exercise (at rest) and during each workload (15 s before the end of every stage); further samples were obtained immediately post-exercise. Duplicate aliquots (100 µL) were immediately deproteinized in 1.0 mL of ice cooled 0.3 M perchloric acid which was then mixed and centrifuged, stored at 70°C, and the extracted supernatant was subsequently analyzed for BLa^–^ using a standard enzymatic spectrophotometric method (Genesys1 –Thermo Fisher Scientific Inc., Waltham, MA, USA).

### 2.9. Statistical Analysis

Data processing and statistical evaluations were completed using Statistica 13.3 software [23] and IBM SPSS Statistics ver. 26.0.0.1 [24]. The characteristics of the studied groups were reported as mean ± SD; 95% confidence intervals (CI) of mean, minimum and maximum values. The level of HR, VO_2_, VCO_2_, RER, BLa^–^, SBP and DBP in the research groups during tests in various workloads are presented in the figures as a mean ± 95% CI. To evaluate the differentiation of HR, VO_2_, VCO_2_, RER, BLa^–^, SBP and DBP, an analysis of variance model with repeated measures of workload (0, 50, 100, 150, and 200 W) and the fixed factor of group (END, STR, and CON) was used. Distributions in samples were compared to normal distribution using the Shapiro–Wilk test. In the case of the studied variables (except for the DBP variable), the distributions in the samples met the criterion of applying the analysis of variance. The homoscedasticity of variance between groups was tested with Bartlett’s test [25], and sphericity of variance for repeated measures with Mauchly’s test [26]. In the case of lack of sphericity of variance, the epsilon Greenhouse–Geisser or Huynh–Feldt correction and the corrected values of the Fisher–Snedecor function were used. If significant differences were found between the studied groups, the differentiation of individual pairs of variables was tested with *post-hoc* Tukey HSD test [23] or Tamhane’s T2 test [27], depending on meeting the assumptions of the variance homoscedasticity. In the analyzed models, the equal size of the effect (η^2^—eta-squared) was calculated, estimating the amount of variance explained by the model in the experiment [26,28]. The differentiation of DBP was tested using the Kruskal-Wallis test, Friedman test and Dunn *post-hoc* test [24]. Statistical significance was set at *p* < 0.05.

## 3. Results

**Figure 1:**
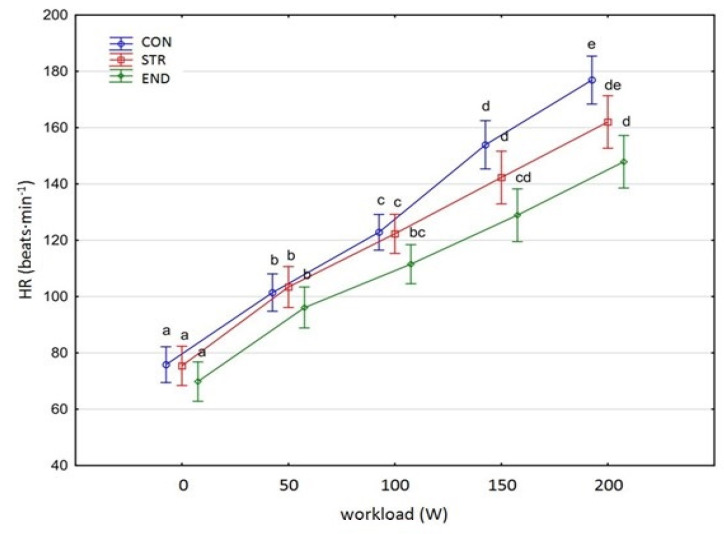
Heart rate (HR) at rest and its response during the progressive exercise test; point and whiskers—mean ± 95% CI; the signs—a, b, c, d, e indicate homogeneous groups (*post-hoc* Tukey’s HSD test: *p* > 0.05).

**Figure 2:**
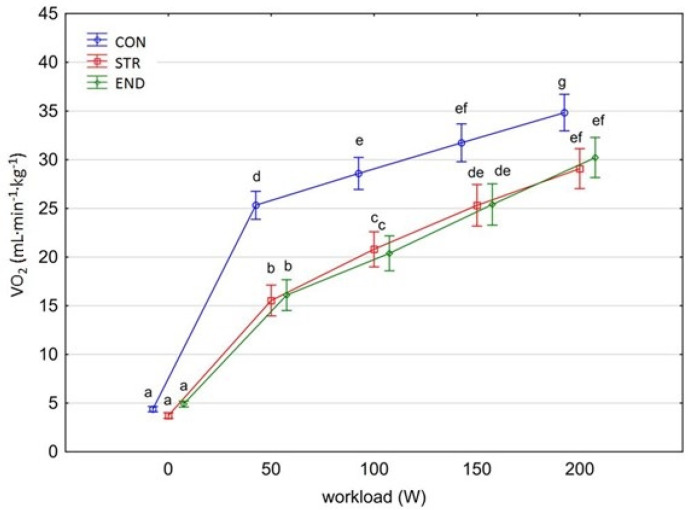
Oxygen uptake (VO_2_) at rest and its response during the progressive exercise test; point and whiskers—mean ± 95% CI; the signs—a, b, c, d, e, f, g indicate homogeneous groups (*post-hoc* Tukey’s HSD test: *p* > 0.05).

**Figure 3:**
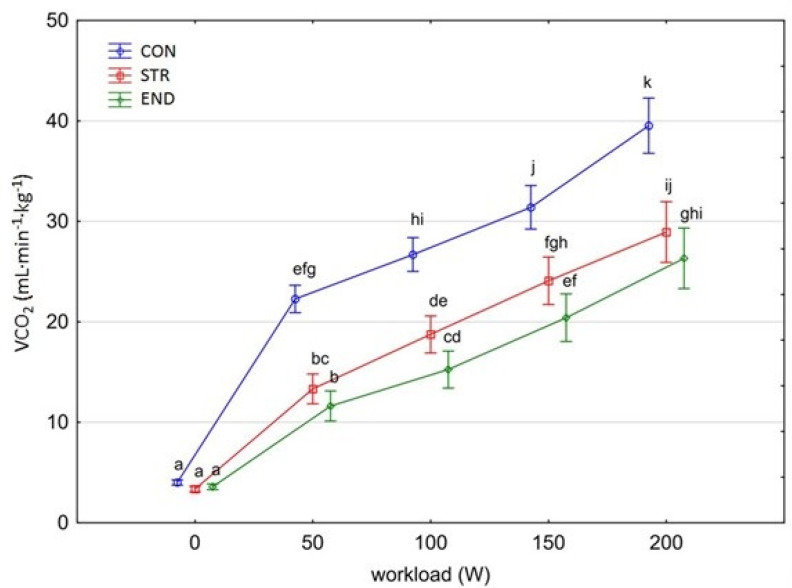
Carbon dioxide production (VCO_2_) at rest and its response during the progressive exercise test; point and whiskers—mean ± 95% CI; the signs—a, b, c, d, e, f, g, h, i, j, k indicate homogeneous groups (*post-hoc* Tukey’s HSD test: *p* > 0.05).

**Figure 4:**
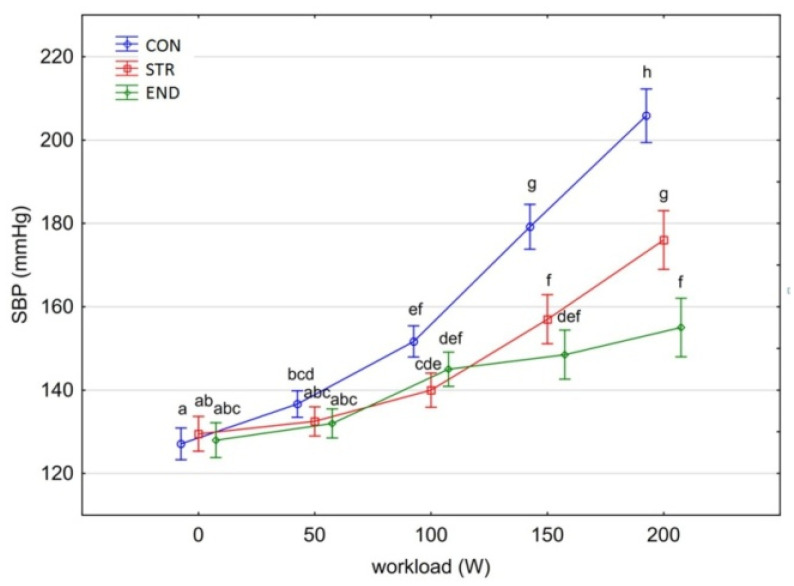
Systolic blood pressure (SBP) at rest and its response during the progressive exercise test; point and whiskers—mean ± 95% CI; the signs—a, b, c, d, e, f, g, h indicate homogeneous groups (*post-hoc* Tukey’s HSD test: *p* > 0.05).

**Figure 5:**
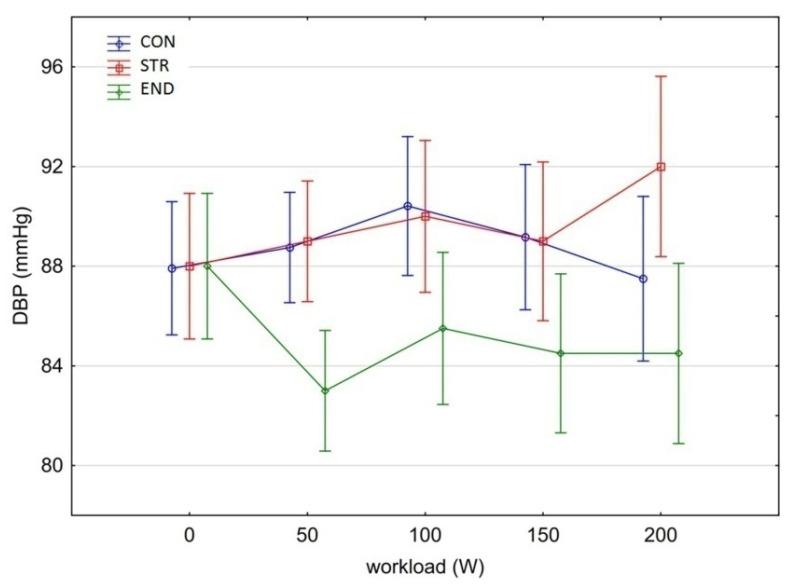
Mean values ± 95% CI of diastolic blood pressure (DBP) at rest and its response during the progressive exercise test.

**Figure 6:**
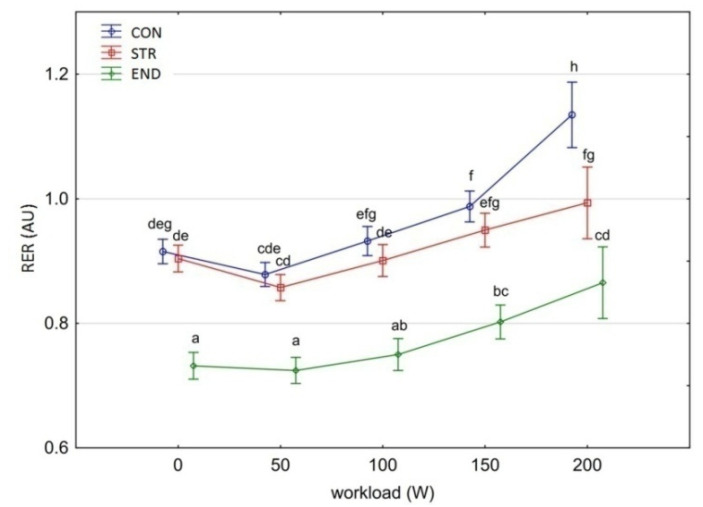
Respiratory exchange ratio (RER) at rest and its response during the progressive exercise test; point and whiskers—mean ± 95% CI; the signs—a, b, c, d, e, f, g, h indicate homogeneous groups (*post-hoc* Tukey’s HSD test: *p* > 0.05).

**Figure 7:**
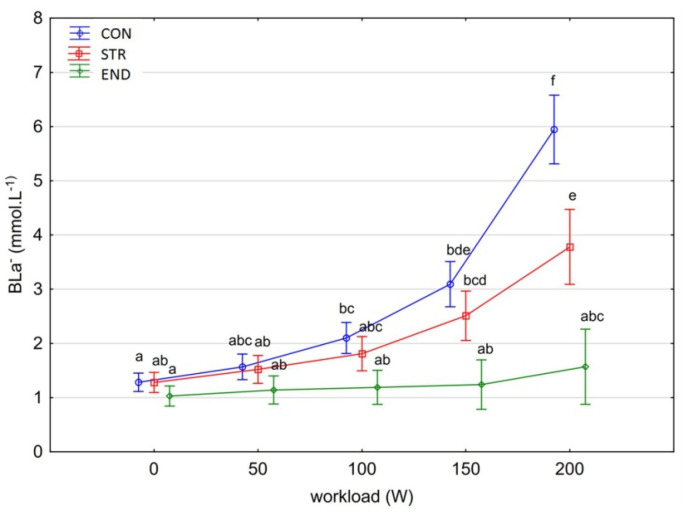
Blood lactate (BLa^–^) at rest and its response during the progressive exercise test; point and whiskers—mean ± 95% CI; the signs—a, b, c, d, e, f indicate homogeneous groups (*post-hoc* Tukey’s HSD test: *p* > 0.05).

Figure 1, Figure 2, Figure 3, Figure 4, Figure 5, Figure 6 and Figure 7 present the comparisons between the mean values of cardiorespiratory and metabolic variables (HR, VO_2_, VCO_2_, SBP, DBP, RER and, BLa^–^) obtained at rest and at the four submaximal workloads (50, 100, 150 and 200 W) of thethree groups.

The training specific adaptation responses, except SBP and DBP, had a similar pattern to thatexpressed in Figure 1, Figure 2, Figure 3, Figure 6 and Figure 7. A significant group×workload interaction effect was shown for HR [F_(8,116)_ = 5.469, *p* < 0.001, η^2^_p_ = 0.274] (Figure 1). The two-way ANOVA demonstrated main effects for group (F_(2,29)_ = 6.623, *p* = 0.004, η^2^ = 0.314) and for workload (F_(4,116)_ = 566.767, *p* < 0.001, η^2^ = 0.951). There was a significant increase in the HR values at subsequent workloads across all groups (G-G-epsilon = 0.483, *p* < 0.001). Significant differences in HR values were shown in all groups between each subsequent workload (Tukey’s test HSD: END, *p* < 0.05; STR, *p* < 0.001; CON, *p* < 0.001).

A significant group×workload interactions effect was shown for both VO_2_ and VCO_2_ [F_(8,116)_ = 21.779, *p* < 0.001, η^2^_p_ = 0.600 and F_(8,116)_ = 17.432, *p* < 0.001, η^2^_p_ = 0.546, respectively]. The level of VO_2_ and VCO_2_ increased significantly at each subsequent workload across groups (for consecutive workload comparisons; Tukey’s HSD test; VO_2_: *p* < 0.001, *p* < 0.001, *p* < 0.01; VCO_2_: *p* < 0.05, *p* < 0.001, *p* < 0.001, respectively) (Figure 2 and Figure 3). However, multiple comparisons between END and STR groups at rest and at each given workload showed no significant differences (*p* = 0.870). A significantly higher level of VO_2_ and VCO_2_ was found in the CON group in comparison with the athlete groups at each workload (Tamhane *T*^2^ test, VO_2_: CON vs. END, *p* = 0.0003; CON vs. STR, *p* < 0.0001; VCO_2_: CON vs. END, *p* <0.0001; CON vs. STR, *p* <0.0001, respectively), but not at rest. No differences were observed between the athlete groups (Tamhane *T*^2^ test, VO_2_: STR vs. END, *p* = 0.959; VCO_2_: STR vs. END, *p* = 0.166).

There was a significant main effect for group in the average SBP [F_(2,29)_ = 37.561, *p* < 0.001, η^2^_p_ = 0.721].The average values of SBP were significantly different between the CON group and the athlete groups (Tukey’s HSD test: CON vs. STR, *p* = 0.0001; CON vs. END, *p* = 0.0001), while there were no differences between them (Tukey’s HSD test: END vs. STR, *p* = 0.072).

The variation of SBP in the studied groups depended on the workload level [F_(4,116)_ = 307.062, *p* < 0.001, η^2^_p_ = 0.914].In the groups of athletes, there were no significant differences at rest, the SBP did not increase significantly with the workload of 50 W (Tukey’s HSD test: *p* = 0.991), and in the STR group also with the workload of 100 W (Tukey’s HSD test: *p* > 0.444) (Figure 4). The increase in workload, 150 W and 200 W, significantly increased SBP in the END and STR groups compared to SBP measured at lower workloads (Tukey’s HSD test: *p* < 0.003). Systolic blood pressure was generally higher in the STR group and increased significantly with increased workloads (150 W vs. 200 W: Tukey’s HSD test: *p* = 0.0001). The values of SBP in the END group did not differ significantly at higher workloads (HSD Tukey’s test: 100 W vs. 150 W, *p* = 0.998; 150 W vs. 200 W, *p* = 0.683).

In the CON group, SBP increased significantly with each increase in workload and the differences in SBP values for subsequent compared pairs of variables were statistically significant (Tukey’s HSD test: *p* < 0.05) (Figure 5).

There was a significant main effect for group in DBP [Kruskal–Wallis test: H (2, N = 160) = 24.650 *p* < 0.00001]. Significant differences in the mean DBP observed across all workloads were found between the END (85.1 mmHg) and STR groups (89.6 mmHg) (Dunn test: END vs. STR, *p* < 0.001) as well as the END and the CON groups (88.8 mmHg) (Dunn test: END vs. CON, *p* = 0.002). There were no differences between the CON and STR groups (Dunn test: CON vs. STR, *p* = 0.570). DBP did not change due to increased workload (Friedman-test: chi sq = 4.198, *p* = 0.380).

There was a significant main effect for group in the average RER [F_(2,29)_ = 101.560, *p* < 0.001, η^2^_p_ = 0.875]. Pairwise comparisons demonstrated a significantly lower RER at rest and at all workloads for the END group compared to the other groups (Tukey’s HSD test: END vs. STR, *p* = 0.0001; END vs. CON, *p* = 0.0001) (Figure 6). There was no difference in RER between the CON and STR groups in the workload from baseline to 150 W (Tukey’s HSD test: *p* > 0.946). The RER at 200 W was significantly higher in the CON group (Tukey’s HSD test: *p* = 0.0001). RER differences in all groups also depended on the workload level [F_(4,116)_ = 76.910, *p* < 0.001, η^2^_p_ = 0.726].

There was a significant main effect for group in the average BLa^–^ [F_(2,29)_ = 33.5999, *p* < 0.001, η^2^_p_ = 0.699]. Post-hoc testing showed significant between-group differences (Tukey’s HSD test: CON vs. STR, *p* = 0.012; CON vs. END, *p* = 0.0001; END vs. STR, *p* = 0.0003). The differences in BLa^–^ also depended on the workload level [F_(4,116)_ = 33.599, *p* < 0.001, η^2^_p_ = 0.699]. At workloads at rest and 50 W, no differences in BLa^–^ values were found between the studied groups (Tukey’s HSD test: *p* > 0.978). At workloads 100 W and 150 W, the level of BLa^–^ was significantly higher in the CON compared to the END group (Tukey’s HSD test: *p* < 0.001). At a workload of 200 W, the differences were significant between all groups (Tukey’s HSD test: *p* < 0.001). BLa^–^ values did not change under workloads in the END group (Tukey’s HSD test: *p*= 0.893), while increased in the STR and CON groups (Figure 7). However, the increase in BLa^–^ due to the increased workloads was higher in the CON group. Most importantly, the interaction effect was significant [F_(8,116)_ = 7.886, *p* < 0.001, η^2^_p_ = 0.658].

## 4. Discussion

In this study, the assessment of the acute cardiorespiratory and metabolic responses was performed on groups of well-trained athletes and recreationally active young men exercising in a laboratory setting at four different submaximal intensities. The substantive finding of this study was that endurance and strength trained athletes differed only in their metabolic responses of RER and BLa^–^ to this incremental cycling test, whereas the acute cardiorespiratory variables did not manifest any statistically relevant differences.

### 4.1. Acute Respiratory Responses

Data from a pooled athletes’ sample (strength and endurance trained) obtained during incremental testing showed that resting and maximal exercise HR were 69.7 ± 7 beats∙min^–^^1^ and 183.5 ± 12.7 beats∙min^–^^1^, respectively [29]. In the current study, across groups, the peak HR (200 W) ranged from 147.9 to 177.4 beats∙min^–^^1^ (corresponding to 74–90% of the age-based predicted maximal HR). This HR range, and the VCO_2_ and RER results provide confidence that the final stage of the cycling exercise (200 W) was achieved with a significant anaerobic contribution. It was expected that HR kinetics should be faster in the STR than in the END athletes. However, in this study, the observed HR trend was similar in both groups. The higher HRs at all submaximal workloads in the STR group may be explained by greater sympathetic nerve activity during exercise, which has been previously reported in strength trained athletes [30]. Generally, as recently demonstrated by Iellamo et al. [30], cardiac autonomic nervous system adaptations to strength training in top-level athletes are dose-related and on an individual basis. These responses are substantially different from those observed in endurance trained athletes and show a progressive shift toward a parasympathetic predominance as training load increases.

In this study, the highest VO_2_ obtained in STR group was 29.1 ± 3.31 mL∙kg^–1^∙min^–1^ and was comparable to the VO_2_ obtained by endurance trained athletes. Thus, on a group basis, the individuals from the END group could not strictly be characterized as top-level endurance triathletes because they demonstrated VO_2_ = 30.2 mL∙kg^–1^∙min^–1^ at 200 W workload. Previous studies showed that Ironman male triathletes performed exercise with mean VO_2max_ values ranging from 52.4 to 72.0 mL∙kg^–1^∙min^–1^ [31]. According to the study’s hypothesis, it was considered that the different chronic training regimes of END and STR influence VO_2peak_ and VO_2_ kinetics differently. It was demonstrated previously that strength and power trained athletes have slow VO_2_ kinetics in comparison with endurance trained athletes [32]. Lower values of VO_2max_ are also typically reported in bodybuilders versus endurance trained athletes (*p* < 0.01) [33]. However, the lack of significant differences in cardiorespiratory function during the submaximal test protocol between the athlete groups, as demonstrated by absolute and relative VO_2_ and VCO_2_ kinetics, peak HR, RER, and SBP, is partly in contrast withother investigations. In particular, the comparable VO_2_ and VCO_2_ kinetics between END and STR groups was not anticipated as it has often been reported in the literature that aerobic fitness is positively correlated to faster overall VO_2_ kinetics [34,35] and that endurance training results in faster overall VO_2_ kinetics [16,35]. It is proposed that in our triathletes there was an increased proportion of type IIA fibers and a reduced proportion of type IIX fibers, elevated maximal muscle strength and increased rapid force capacity [15,36]. It is possible that the cycling economy may already be highly optimized in top-level cyclists or triathletes and therefore highly difficult to improve. The relative VO_2peak_ attained by STR athletes (comparable with END athletes) might result from their style of bodybuilding with heavy resistance training versus that of a competitive weight lifter. Interestingly, this type of training (combined with endurance training) was found to enhance long-term endurance capacity (improved 45-min time trial performance) in highly-trained cyclists (VO_2max_ 71–75 mL∙kg^–1^∙min^–1^) [37]. Moreover, strength training may provide a stimulus for microvascular adaptations such as: capillary neoformation, changes in morphology, and increased capillary density [38]. Another alternative explanation might be the similar respiratory muscle adaptations in END and STR groups (i.e., maximal inspiratory and expiratory pressure). For instance, Hacket et al. [33] demonstrated the evidence of greater respiratory muscle strength in bodybuilders compared to endurance-trained athletes.

During training and/or competition, endurance trained athletes (including triathletes) sustain long work intervals with high CO, HR, SV and a moderate increase in mean arterial BP [39]. The latter is explained due to dynamic and static load components. On the other hand, strength training is associated with a marked elevation in SBP and DBP [40]. Interestingly, the BP response during weightlifting can increase to levels as high as 320/250 mmHg [41]. In addition, high BP response to exercise predicts the future development of hypertension in young athletes [42].

Currently, there appears to be little consensus in the literature regarding the clinical relevance of assessing the BP during physical exercise, and the normal values and upper limits of BP response to exercise are not well defined in highly trained athletes [43]. Nevertheless, although we observed statistically significant differences in mean SBP between the CON group and both groups of athletes, there was no differentiation between END and STR groups. In addition, contrary to STR and CON groups, SBP in the END group did not differ significantly with increasing workload. However, in this study the observed BP responses across the groups are well recognized [44]. Interestingly, Colliander and Tesch [45] found that SPBs and DBPs at rest and SBP response during progressive cycle ergometer exercise at 100–200 W were comparable in age-matched bodybuilders and medical students. Bodybuilders, however, displayed lower (*p* < 0.01–0.001) HR at identical power outputs of exercise.

### 4.2. Acute Metabolic Responses (RER and BLa^–^)

In the case of the analysis of metabolic changes, we know that RER indirectly shows the muscle’s oxidative capacity [46]. During high-intensity efforts, the RER level usually exceeds 1.0 AU. In this study, the most evident between-group differences were found in metabolic values (RER and BLa^–^). Specifically, it was observed in the recreationally active participants that the peak RER value (at workload of 200 W) exceeded 1.0 AU, while the best adaptation, the lowest RER, to the submaximal workloads was observed in the END group. However, there was a significant between-group difference in resting RER which is hard to explain and might be caused by the greater pre-exercise stress in individuals from STR and CON groups. In a few previous studies, comparable results in trained, low-trained, and untrained individuals have been found [46,47]. Similar to HR response, peak RER values ≥ 1.0 AU (in CON group) and ~1.0 AU (STR group) also indirectly suggest that the final stage of cycling exercise was mainly performed in anaerobic conditions. Goedecke et al. [48] observed the large variability in RER at rest and during exercise at different intensities in endurance-trained athletes. Wade et al. [49] found that RER during mild exercise (100 W) was inversely associated with the proportion of type I (slow twitch) muscle fibers. Moreover, the RER values obtained in professional endurance-trained athletes (cyclists) were lower than 1.0 at workloads less than 400 W during pre-competition and competition periods [50]. In general, sedentary lifestyle increases the RER values, whereas physically active and trained subjects exhibit lower RER than untrained subjects in response to comparable workloads [46,51]. Endurance training also decreases the RER values, so this would partly explain the observed differences in RER during submaximal exercise between END, STR, and CON groups.

According to Ramos-Jiménez et al. [46], RER during submaximal exercise is associated with other variables (HR_max_, VO_2max_, workload, and lactate threshold). For instance, Wade et al. [49] report that during high-intensity exercise (~70% W_peak_) circulating plasma lactate concentration contributed significantly to the model predicting RER. While questions remain regarding the cause–effect relationships among these variables we have observed that average and peak BLa^–^ were significantly lower in the END group compared to STR and CON groups. According to previous studies, one of the main metabolic adaptations to aerobic exercise at a muscular level is less BLa^–^ production in trained compared with untrained participants during exercise at a given intensity or exercise workload [52]. This adaptation was observed in our study where BLa^–^ was remarkably lower (2–4-fold) and statistically different in the END group compared with both the STR and CON groups throughout exercise. Indeed, we found that, along with RER, BLa^–^ levels differed between each exercise intensity.This agrees with the results presented by Gollnick et al. [53] who found that athletes with a predominance of fast-twitch motor units have been shown to produce twice as much lactate compared with athletes with a predominance of slow-twitch motor units. However, they compared the alterations in lactate metabolism during maximal exercise test. In contrast, previous studies on highly trained endurance athletes (cyclists) showed that during cycle ergometer testing, they produced comparable BLa^–^ to non-cycling trained males exercising at different workloads [54]. Nevertheless, the fiber-type characteristics may account for differences in the amount of BLa^–^ that is formed during exercise for specific types of athletes [55]. The lowest BLa^–^ observed in the END group was most probably due to their specific training adaptations that increase BLa^–^ clearance capacity. During high-intensity exercise, lactate production and BLa^–^ appearance rate is greater in trained than in lower-trained or untrained individuals [56]. Moreover, due to low adaptation to exercise the relatively high BLa^–^ observed at the end of the cycling test, especially in control individuals, could also be interpreted as a manifestation of fatigue. Overall, the findings of the present study suggest that the heightened adaptation of processes affecting lactate metabolism may be more influential during a short-term cycling exercise test in endurance-trained athletes compared to strength-trained and recreationally active individuals.

### 4.3. Limitations

Potential limitations of this study should be acknowledged. Firstly, it has a relatively small sample size. Secondly, although our participants were competitive athletes with at least 4–5 years of training history, they might be described as sub-elite rather than elite given that they competed at regional and national rather than international level. Thirdly, it was not possible to reconstruct and compare the detailed training regimes, let alone the training load calculated, e.g., as volume (min) × intensity (%HR_max_ or %1RM) of END and STR groups over the past years, thus there may be great variability in training stimulus across athletes. Fourthly, athletes from the END group were triathletes, so they routinely performed cycle exercise training. Hence, the type of exercise test was more specific for them, while for the athletes from STR group it was the nonfamiliar mode. Fifthly, the study involved young men, so the ability to generalize the results/conclusions to people of other genders and ages is limited. Finally, it could be possible that the duration of the cycling test, according to our protocol (not to exhaustion), may not fit the time needed to obtain in-depth physiological and metabolic analysis in endurance- and strength-trained athletes, therefore a longer version of the protocol could be adequate.

## 5. Conclusions

In conclusion, this study revealed that acute cardiorespiratory responses at each of the four submaximal exercise efforts were significantly lower in endurance trained athletes compared to strength trained or recreationally active men. However, in the two athletically trained groups significant differences in metabolic responses in both RER and BLa^–^ were shown. RER analysis can help identify physical fitness status at low exercise intensity.The results from this research supported the view that peripheral metabolic adaptations are the main cause of the lowerlactatelevels observed after endurance training. Simultaneously, the cross-sectional design of this study precluded any strong conclusions regarding the possible mechanisms by which long-term endurance training may lower RER.HR and RER responses observed in STR and CON groups indirectly suggest that the final stage of cycling exercise (workload of 200 W) was mainly performed in anaerobic conditions. The larger increases in cardiorespiratory and metabolic parameters at every time point (given workload or exercise intensity) observed in the CON group may relate to their lower physiological profile and substantially greater room for improvement compared to END and STR groups. For endurance-trained athletes, we propose to follow concurrent training programs (combined strength and endurance training) to improve neuromuscular parameters and thus optimize their economy of movement and endurance-specific muscle power factors.

## Figures and Tables

**Table 1:** The characteristics of the studied groups.

Group	Variable	Mean ± SD	95% CI	Median	Min	Max
END (*n* = 10)	Age (years)	20.3 ± 0.62	18.9–21.7	19	19	24
	BH (cm)	179.8 ± 2.08	175.1–184.5	181	165	190
	BM (kg)	75.06 ± 2.935	66.42–81.70	80.5	59.2	83.5
	BMI (kg/m^2^)	23.16 ± 0.644	21.70–24.62	23.3	18.9	25.9
STR (*n* = 10)	Age (years)	22.4 ± 0.43	21.4–23.4	22	20	24
	BH (cm)	181.9 ± 2.40	176.5–187.3	179	173	192
	BM (kg)	83.93 ± 2.704	77.81–90.05	82.6	75.0	103.7
	BMI (kg/m^2^)	25.37 ± 0.639	23.92–26.81	25.0	22.8	28.4
CON (*n* = 12)	Age (years)	23.2 ± 0.41	22.3–24.2	24	21	25
	BH (cm)	181.6 ± 1.82	177.6–185.6	184	173	189
	BM (kg)	78.23 ± 2.579	72.56–83.91	78.6	65.0	91.0
	BMI (kg/m^2^)	23.70 ± 0.635	22.30–25.10	23.6	20.6	28.7

Notes: END—endurance trained athletes; STR—strength trained athletes, CON—control group; BH—body height; BM—body mass; BMI—body mass index; SD—standard deviation; 95% CI—95% confident intervals of mean; Min—minimum value; Max—maximum value.

## Data Availability

Data are available upon request from the corresponding author.
